# Factors associated with elevated gentamicin trough levels in neonates: a retrospective analysis of dosing and clinical parameters

**DOI:** 10.3389/fped.2025.1510838

**Published:** 2025-03-17

**Authors:** Julian Trah, Philipp Deindl, Alexandra Luister, Claudia Langebrake, Dominique Singer, Chinedu Ulrich Ebenebe

**Affiliations:** ^1^ Department of Neonatology and Pediatric Intensive Care Medicine, University Children’s Hospital, University Medical Center Hamburg-Eppendorf, Hamburg, Germany; ^2^ Department for Pediatric Rehabilitation, VAMED Rehabilitation Hospital, Geesthacht, Germany; ^3^ Hospital Pharmacy, University Medical Center Hamburg-Eppendorf, Hamburg, Germany

**Keywords:** neonate, gentamicin, aminoglycoside, trough levels, dosing

## Abstract

**Objective:**

Investigate determinants of elevated gentamicin trough levels in neonates.

**Methods:**

This single-center retrospective analysis used a multivariate linear regression model to explore the relationship between gentamicin trough concentrations and factors such as creatinine levels, dosage, day of life, sex, CRP levels, and dosing interval in neonates.

**Results:**

In 215 neonates, including 68 (31.6%) premature neonates with a postmenstrual age of ≤35 weeks, shorter dosing intervals, higher creatinine levels, and increased dosage were linked to higher gentamicin trough levels. Elevated CRP levels corresponded with lower trough levels.

**Conclusion:**

This study highlights the critical role of dosing frequency, kidney function, and inflammatory status in influencing gentamicin trough levels in neonates. However, all gentamicin trough levels were within the 2 µg/ml threshold.

## Introduction

Gentamicin is widely used to treat neonates at risk of infection and those with suspected or confirmed infections (1). Gentamicin and beta-lactam antibiotics are an effective combination therapy against a wide range of pathogens prevalent in the neonatal stage, such as *Streptococcus agalactiae* (group B streptococcus), *Listeria monocytogenes*, *Enterococcus faecalis*, and most isolates of *Escherichia coli* (2). Gentamicin is an antibiotic with a concentration-dependent bacterial killing effect, and the challenge in aminoglycoside therapy lies in optimizing dosages to maximize therapeutic benefits while minimizing risks like nephrotoxicity and ototoxicity (3, 4). Traditionally, a 2.5 mg/kg dose of gentamicin every twelve hours was standard (5). Over the past twenty years, adjustments have been made to increase the dose to 5 mg/kg and to prolong dosing intervals to 24–48 h, tailored according to the neonate's postmenstrual age (6–8). This adjustment aims to optimize bactericidal activity through higher gentamicin peak serum levels (>5 μg/ml) and to reduce toxicity risks by avoiding elevated trough levels (>2 μg/ml) (4, 9, 10). Customizing gentamicin dosages requires therapeutic drug monitoring, mainly focusing on trough concentrations to confirm adequate clearance prior to subsequent dosing. Given the significant variability in gentamicin pharmacokinetics among neonates, attributed to ongoing physiological development and organ maturation affecting distribution volume and kidney clearance, there's a pronounced need to understand individual responses to gentamicin therapy (11). This variability is observed even among neonates of similar gestational and postnatal ages (12). Our research thus seeks to investigate determinants of elevated gentamicin trough levels in this population, aiming to refine dosing strategies for improved safety and efficacy.

## Materials and methods

### Study design and subjects

The study was a retrospective cohort involving neonatal patients treated with gentamicin (Ratiopharm, Ulm, Germany) within their first week of life at our tertiary perinatal center from August 2020 to January 2022. The Hamburg Medical Association Ethics Committee approved the study with waived informed consent (2022-300189-WF).

Our institutional gentamicin regimen within the first week of life comprises dosing of 4 mg/kg and an interval of 48 h, 36 h, and 24 h in neonates with a postmenstrual age of <30 weeks, ≥30–34 + 6 weeks, and ≥35 + 0 weeks, respectively (13). Gentamicin trough concentrations were measured before administering the third dose. Subjects were excluded if dosing intervals differed>10% from the standard interval or the time of trough concentration measurement was inaccurate. Our clinic's target gentamicin trough concentration is <1 µg/ml and independent of isolated organisms (14, 15).

Clinical patient data were obtained by reviewing the hospital's healthcare information systems (Soarian®, Siemens Healthcare, Erlangen, Germany; ICM®, Draeger, Luebeck, Germany). Extracted information included sex, weight, day of life and postmenstrual age at treatment initiation, absolute and relative (per kg body weight) gentamicin dose, dose interval, gentamicin trough concentrations, highest C-reactive protein (CRP) concentration during gentamicin treatment, and creatinine and urea concentrations at the time of gentamicin trough concentration measurement. Only the first episode was included in the analysis to exclude cluster effects in patients who underwent multiple gentamicin treatments.

Gentamicin concentrations were measured by a homogeneous particle-enhanced turbidimetric inhibition immunoassay (PETINIA) (Atellica CH Gentamicin, Siemens Healthcare, Erlangen, Germany), creatinine concentrations were analyzed using the photometric modified Jaffe method (Atellica CH Creatinine, Siemens Healthcare, Erlangen, Germany), the urea concentration was determined via photometric detection, and CRP was analyzed with immunoturbidimetry (Atellica CH C-Reactive Protein, Siemens Healthcare, Erlangen, Germany).

### Statistical analysis

Statistical analysis was performed using SPSS, version 29 (IBM, Armonk, NY, USA) and R Version 4.2.0 (R Core Team, Vienna, Austria). Data on neonatal demographics were expressed as the mean and standard deviation (SD) for continuous variables and counts and percentages for categorical variables. We calculated a multivariate linear regression model including the continuous predictors, maximum creatinine concentration, applied gentamicin dose per body weight, day of life, and the categorical predictors, sex, elevated CRP, and the dosing interval to analyze the impact on the gentamicin trough concentration after ruling out multicollinearity between predictors (16).

Variables were collected retrospectively based on clinical relevance and prior evidence, and a multivariate regression model was constructed to adjust for confounders. Clinically significant variables were included irrespective of univariate results to ensure comprehensive analysis. The model adhered to an event-to-variable ratio of approximately 1:30, selecting variables judiciously to avoid overfitting and maintain robustness. Model fit and validity were evaluated through adjusted *R*^2^ and residual analysis.

## Results

### Demographic characteristics

Our analysis covered 282 gentamicin treatment instances, from which 67 were excluded due to non-adherence to the inclusion criteria, leaving a cohort of 215 for analysis. Of these, 68 patients (31.6%) were preterm infants with a postmenstrual age of 35 weeks or less. Detailed demographic data and gentamicin dosing information are shown in Table 1.

**Table 1:** Characteristics of the 215 neonates included in the study.

Characteristics	All *n* = 215	Postmenstrual age category, weeks		
		<30 + 0 *n* = 16	≥30 + 0–34 + 6 *n* = 52	≥35 + 0 *n* = 147
Female, *n* (%)	87 (40.5)	8 (50.0)	25 (48.1)	54 (36.7)
Age, days	1.3 (1.1)	0.5 (0.3)	0.7 (0.5)	1.6 (1.2)
Postmenstrual age, weeks	36.9 (4.2)	28.2 (1.5)	32.5 (1.4)	39.3 (1.9)
Weight, g	2,765 (942)	1,069 (231)	1,814 (502)	3,286 (545)
Gentamicin dose, mg/kg	3.84 (0.30)	3.66 (0.40)	3.78 (0.35)	3.88 (0.27)
Dose interval, h	29.0 (7.9)	47.4 (3.1)	35.4 (5.9)	24.8 (2.9)
Gentamicin trough concentration, μg/ml	0.82 (0.46)	0.39 (0.23)	0.75 (0.53)	0.89 (0.42)
Gentamicin trough concentration >1 μg/ml, *n* (%)	69 (32.1)	0 (0)	16 (30.8)	53 (36.1)
C-reactive protein, mg/L	10.5 (19.4)	6.4 (14.6)	3.7 (7.7)	13.3 (22.1)
Creatinine, mg/dl	0.57 (0.25)	0.85 (0.44)	0.64 (0.22)	0.52 (0.21)
Urea, mg/dl	8.1 (8.0)	5.6 (11.2)	10.3 (9.0)	7.6 (7.0)

Values are shown as mean (SD) for continuous variables and as counts (percentage) for categorical variables.

Gentamicin therapy commenced within the first 24 h for 131 patients (61%) and between 24 and 48 h for another 39 patients (18%). The mean gentamicin dosages administered remained slightly below the target dosage of 4 mg/kg in all groups. Among the cohort, 69 patients (32.1%) exhibited gentamicin trough concentrations exceeding 1 µg/ml, with the highest serum levels observed in neonates assigned a 24 hour dosing interval. Notably, average CRP levels were higher in patients ≥35 weeks postmenstrual age compared to the more preterm infants.

### Multivariate linear regression analysis

**Figure 1:**
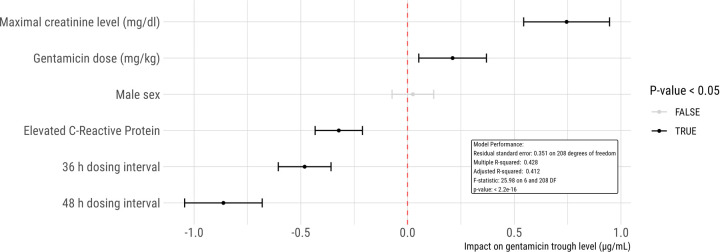
Linear regression model to analyze the impact of potential risk factors on gentamicin trough concentrations. Each dot represents an estimate, accompanied by its 95% confidence interval. In this model, female gender, normal CRP levels, and a 24 h dosing interval serve as the reference standards. For instance, the model predicts that an increase in creatinine by 1 mg/dl would lead to an elevation in gentamicin trough concentration by 0.75 µg/ml.

A multivariate linear regression model was developed to assess the influence of various factors on gentamicin trough concentrations (Figure 1). This model highlighted that dosing intervals of 36 h and 48 h and increased CRP levels significantly correlated with lower gentamicin trough concentrations (*p* < 0.001 for both intervals and CRP elevation). Specifically, gentamicin trough levels above 1 µg/ml were observed in 15% of patients with CRP levels over 10 mg/L, in contrast to 39% of those with CRP levels within the normal range.

Furthermore, the analysis identified higher creatinine levels (*p* < 0.001) and increased gentamicin dosages (*p* = 0.009) as predictors of elevated gentamicin trough levels. A comparison within the cohort revealed that 43% of patients with creatinine levels exceeding 0.6 mg/dl had trough levels above 1 µg/ml, compared to 27% among those with normal creatinine levels.

The regression analysis also determined that neither the patients' sex (*p* = 0.61) nor their age in days at the time of treatment (*p* = 0.34) significantly affected gentamicin trough levels.

## Discussion

Navigating the balance between achieving the maximal bactericidal impact and avoiding toxic levels of gentamicin in neonatal sepsis treatment continues to pose a challenge (3). Several dosing regimens have been suggested for neonates depending on gestational age and weight, ranging from 3 to 7.5 mg/kg (17). Most studies recommend dosages of 4–5 mg/kg, resulting in a mean peak concentration of 5.4–11.2 mg/L. In our study, administered gentamicin dosages across different postmenstrual age groups fluctuated between 3.66 and 3.88 mg/kg (Table 1). O'Connor et al. demonstrated that a gentamicin dosing regimen of 3.5 mg/kg achieved therapeutic peak concentrations (>5 µg/ml) in 98% of the neonates (18).

Our multivariate linear regression analysis underscored that dosing intervals extending to 36 and 48 h significantly contribute to lower gentamicin trough levels compared to a 24 h schedule. Notably, none of the neonates receiving gentamicin every 48 h exhibited trough concentrations above 1 µg/ml. This finding substantiates the efficacy of extended interval dosing adjusted for postmenstrual age in preventing elevated gentamicin trough concentrations in preterm neonates with a reduced drug clearance due to kidney immaturity (19).

For neonates aged 35 weeks or more with 24 h dosing intervals, we observed supratherapeutic trough levels (>1 µg/ml) in 36% of cases, albeit not surpassing 2 µg/ml (maximum 1.9 µg/ml). This aligns with findings by DeHoog et al., who argued that existing dosing algorithms aim to maintain serum trough concentrations below 2 µg/ml rather than 1 µg/ml, given the association of higher concentrations with toxicity (9, 20). Although many centers, such as ours, aim trough levels <1 µg/ml, numerous studies suggest that only gentamicin trough concentrations >2 µg/ml are associated with an increased risk of nephrotoxicity and ototoxicity (21, 22). Therefore, implementing a safe threshold of 2 µg/ml would result in less supratherapeutic levels, leading to unnecessary drug monitoring.

The analysis highlighted a significant link between increased serum creatinine levels and higher gentamicin trough concentrations. This finding is consistent with Antolik et al., who stated that neonates with a gestational age of ≥30 weeks with a creatinine concentration ≥1 mg/dl within the first 12–24 h of life were more likely to have an elevated gentamicin trough concentration (>1 µg/ml) than their counterparts with normal creatinine concentrations (23). As aminoglycosides are almost entirely eliminated by the kidneys, clearance of these drugs is directly affected by the patient's glomerular filtration rate (24). Hence, serum creatine values may play an essential role in identifying neonates at risk for elevated serum gentamicin trough concentrations. Kayser et al. further demonstrated the potential for reducing the incidence of elevated trough levels through a creatinine-based dosing algorithm (15). However, the British National Institute for Health and Care Excellence advises against using serum creatinine for monitoring and dosage adjustments in infants on gentamicin, as creatinine levels can be influenced by various factors, including maternal creatinine, infections, and conditions like hypoxic-ischemic encephalopathy (12, 25).

Previous studies in critically ill adults and children indicate that the volume of distribution of gentamicin increases during sepsis (26–28). Lingvall et al. reported a 14% increase in distribution volume in septic neonates, suggesting larger doses may be necessary to achieve therapeutic peak levels in the presence of sepsis (29). This study's finding that higher CRP levels, indicative of sepsis, correlate with lower gentamicin trough concentrations underscores the importance of careful gentamicin use in neonates with suspected infections, especially since those with lower CRP levels are more prone to elevated trough concentrations.

### Limitations

This study's retrospective single-center nature and adherence to institutional treatment protocols may limit the generalizability of our findings. The impact of potentially nephrotoxic medications on different patient groups was not examined. Also, we did not investigate whether elevated gentamicin trough levels were associated with drug toxicity. We acknowledge that the relatively small sample size and limited number of outcome events may reduce the generalizability and robustness of the multivariate regression model. Furthermore, the potential for residual confounding cannot be excluded, as certain clinically relevant variables not included in the dataset may have influenced the observed relationships.

## Conclusion

Our findings suggest that a 24 h dosing interval, elevated creatinine levels, and lower CRP levels are linked to higher risks of gentamicin concentrations >1 µg/ml. However, all gentamicin trough levels were within the well-established 2 µg/ml threshold.

## Data Availability

Requests to access these datasets should be directed to jtrah@uke.de.
