# Supplementary material for: Factors associated with elevated gentamicin trough levels in neonates: a retrospective analysis of dosing and clinical parameters
Source: Front Pediatr. 2025 Mar 17;13:1510838. doi: 10.3389/fped.2025.1510838 (PMC11956741; doi:10.3389/fped.2025.1510838)
Supplement: Supplementary file 1 [file Datasheet1.pdf]

Supplementary Material: Multivariate linear regression model

A multivariate linear regression model was employed to estimate the precise impact of individual factors on the measured gentamicin trough levels, deliberately avoiding a logistic regression model due to the uncertain data regarding the upper gentamicin threshold. Variables were selected retrospectively based on clinical relevance and prior evidence. Clinically significant variables, such as CRP levels, dosing intervals, and creatinine levels, were included irrespective of univariate significance to ensure a comprehensive evaluation. The model adhered to an event-to-variable ratio of approximately 1:30, minimizing the risk of overfitting and maintaining robustness.

To assess multicollinearity, Variance Inflation Factors (VIFs) and tolerance values were calculated, confirming no concerning levels of collinearity. The results are summarized in the table below. The residuals of the model were evaluated for normality using the Shapiro-Wilk test: (W = 0.98856, p = 0.08383), and their distribution was visualized in a Q-Q plot, included in Figure 1.

Variable	Tolerance	VIF
Male Sex	0.9702182	1.030696
Gentamicin Dose (mg/kg)	0.9518869	1.050545
Elevated CRP	0.8613455	1.160974
36 h Dosing Interval	0.8183372	1.221990
48 h Dosing Interval	0.8001851	1.249711
Maximal Creatinine Level	0.8559910	1.168237

The goodness-of-fit of the model was further assessed via adjusted R^2 and residual analysis, ensuring the model's validity: Residual standard error: 0.3513 on 208 degrees of freedom, Multiple R-squared: 0.4284, Adjusted R-squared: 0.4119, F-statistic: 25.98 on 6 and 208 DF, p-value: < 2.2e-16. The table below shows the detailed results of the regression model for each variable.

Coefficients	Estimate	Std. Error	t value	Pr(>|t|)	Significance
**(Intercept)**	-0.14586	0.32769	-445	0.6567	
**Male Sex**	0.02518	0.04955	508	0.61184	
**Gentamicin Dose (mg/kg)**	0.21121	0.08039	2.627	0.00925	**
**Elevated CRP**	-0.32209	0.0562	-5.731	3.47e-08	***
**36 h Dosing Interval**	-0.48203	0.06268	-7.69	5.77e-13	***
**48 h Dosing Interval**	-0.86265	0.0922	-9.357	< 2e-16	***
**Maximal Creatinine Level**	0.74544	0.10205	7.304	5.84e-12	***

Despite these measures, we acknowledge limitations, including the relatively small sample size and restricted number of outcome events, which may affect the model's generalizability. Additionally, potential residual confounding from variables not captured in the dataset remains a possibility. These limitations have been explicitly addressed in the

manuscript and supplementary material, with recommendations for larger, multi-center studies to validate our findings.

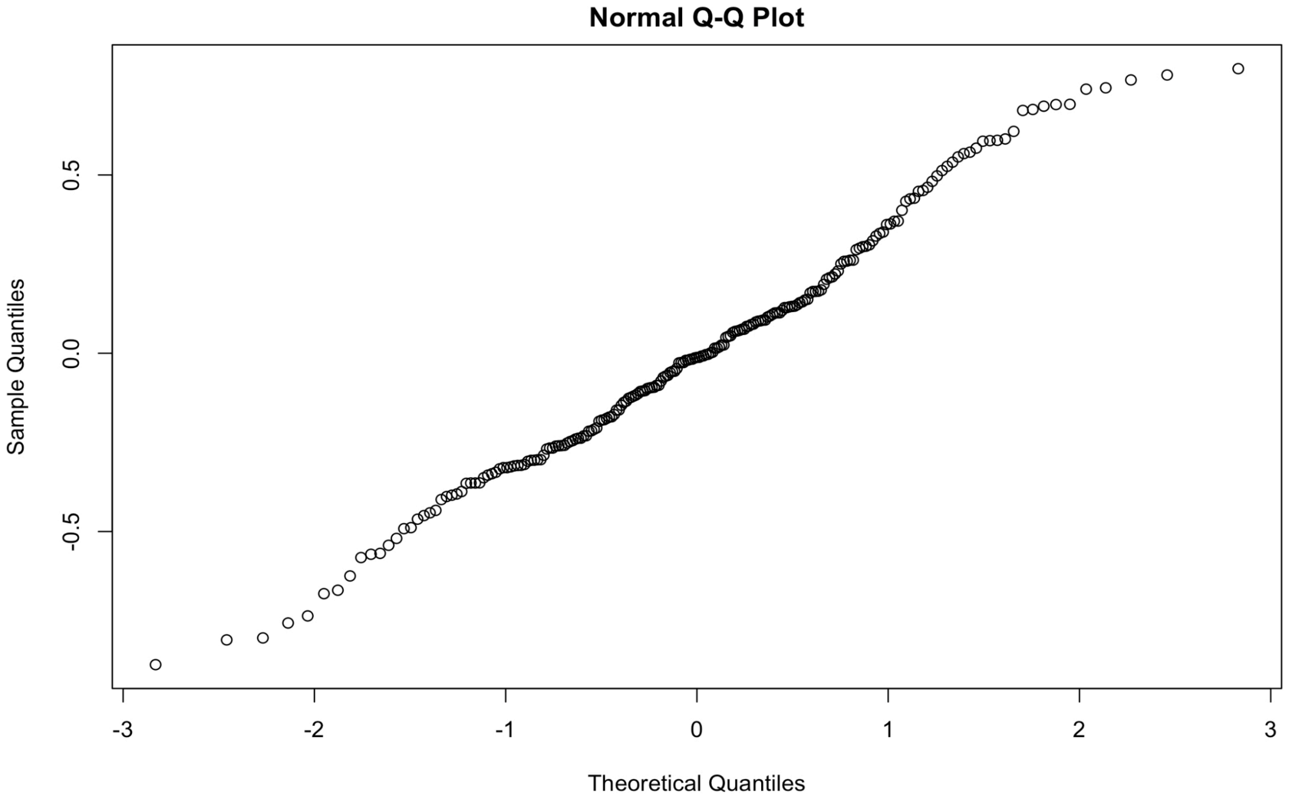

Figure 1: The Q-Q plot illustrates the normality of the residuals from the multivariate linear regression model. Each point represents a standardized residual plotted against the expected quantiles of a normal distribution.
